# Qualitative Evaluation of a Novel Security Role to Reduce Occupational Violence in Inpatient Hospital Settings

**DOI:** 10.1177/21650799241249187

**Published:** 2024-05-30

**Authors:** Jed Duff, Lita Jeffries, Joanna Griffiths, Kaylene Woollett, Andy Carter, Hui (Grace) Xu

**Affiliations:** 1 School of Nursing, Centre for Healthcare Transformation, Queensland University of Technology; 2 Nursing and Midwifery Research Centre; 3 Royal Brisbane and Women’s Hospital Queensland Occupational Violence Strategy Unit, Royal Brisbane and Women’s Hospital

**Keywords:** Ambassador, inpatient wards, occupational violence, security service models, security officer

## Abstract

*Background:* Occupational violence (OV) is a priority issue that significantly affects the safety of nurses, leading to staff burnout and poor retention issues. Security personnel are common in inpatient settings, yet there is limited research on their role, function, and impacts. The study aims to qualitatively evaluate a novel security role to reduce OV in inpatient settings. *Methods*: This qualitative study was conducted in a tertiary hospital in Brisbane, Queensland. A novel security role (Ambassador) was piloted in three inpatient wards over 6 months in 2020 to mitigate OV risk. Semi-structured in-depth interviews were conducted. All interviews were audio recorded. Interview transcripts were transcribed. Deductive analysis based on the Consolidated Framework for Implementation Research (CFIR) was used to identify the experiences and perceptions of the participants. *Findings*: 17 participants were interviewed. Five themes were identified including program characteristics, internal drivers, external drivers, individual experience, and implementation process. An Ambassador proactively engages with patients and visitors and employs behavioral strategies to de-escalate or redirect persons of concern. Participants considered Ambassadors to be important members of the health care team who supported the provision of patient and family-centered care. Successful implementation was said to require collaboration between clinical and security services and a small agile project team with authority and autonomy. *Conclusion/Application to practice*: This study provides many insights into the successful implementation of a novel security role in acute hospital settings. More research is needed on the effectiveness, appropriateness, feasibility, and cost of different security models.

## Background

Occupational Violence (OV) in health settings is defined as an incident where an employee is abused, threatened, or assaulted by patients, consumers, relatives, and friends of a patient, or members of the public, irrespective of the intent for harm (Queensland Health, 2016). OV in health care settings is a significant problem globally, with increasing violence directed toward health care staff, especially nurses (Lawrence et al., 2018; Muir-Cochrane et al., 2020). A systematic review found that globally, 62% of health care workers reported exposure to workplace violence (J. Liu et al., 2019). Nurses are the health care workers at the highest risk of exposure to OV because they provide 24-hour, direct patient care. Reports indicate that up to 89% of nurses have experienced verbal or physical assault (Bernardes et al., 2021; Wressell et al., 2018). Ninety percent of these incidents were preventable (Bernardes et al., 2021). OV has significant physical, psychological, social, and economic impacts on the health care workforce and community (Mento et al., 2020). Studies demonstrated a direct correlation between OV with low job satisfaction, burnout, absenteeism and high job turnover rate (Kafle et al., 2022).

The use of security personnel to mitigate risk or manage aggressive behavior is frequently reported, mainly in mental health services and emergency departments (Lawrence et al., 2018; Muir-Cochrane et al., 2020). The roles and functions of security personnel vary greatly but generally include controlling the admission of visitors and patients, monitoring high-risk patients, restraining and secluding patients, and monitoring closed-circuit televisions (Schoenfisch & Pompeii, 2016; Shannon et al., 2015). One study has highlighted that security staff can act as interpersonal contact for patients (Schoenfisch & Pompeii, 2016), which reflects the important role of security as supporting personnel.

Preventing and managing OV is a key priority globally for health services in various settings, including the Australian Queensland State Government, as increasing violence is directed toward health care staff (Queensland Health, 2016; Schoenfisch & Pompeii, 2016). (Queensland Health, 2016). The Queensland Occupational Violence Strategy Unit (QOVSU) is a statewide unit that develops, implements, scales, and spreads OV prevention initiatives throughout Queensland. In addition, Statewide Unions such as the Queensland Nurses and Midwives Union (QNMU) have prioritized OV prevention and actively lobbied for the implementation of prevention strategies at the facility, health service, and government levels (Queensland Nursing and Midwifery Union, 2023).

Ambassadors are non-traditional security personnel embedded in at-risk environments that use a proactive client-centered approach to OV prevention and management. The novel role was developed based on the Canadian Client Services Ambassador model, which demonstrated a reduction in the incidence of violence by 40% in emergency departments (Young, 2017). In Queensland, Australia, the role had been piloted in emergency departments and a residential aged care facility with positive outcomes including significantly lower OV incident rates and high staff satisfaction (Jokic, 2022; WorkCover Queensland, 2022). However, it had not previously been used in acute inpatient wards. There has been no previous study exploring the use of a proactive security model to reduce OV against health care staff in inpatient settings. Therefore, this study aims to provide a comprehensive qualitative description of the Ambassador program, which will be essential for future implementation and spread.

## Methods

### Design

This interpretive descriptive qualitative study (Ghorbani & Matourypour, 2020) adopted a pragmatic approach to describe participants’ subjective experiences and perceptions to generate insights into this novel role and its implementation. The study has been reported using the consolidated criteria for reporting qualitative research (COREQ; Tong et al., 2007). Ethical approval was obtained from the hospital’s Human Research Ethics Committee (HREC/2021/QRBW/74335).

### Analytical Framework

The Consolidated Framework for Implementation Research (CFIR) was used to analyze and present the findings of the study (Damschroder & Lowery, 2013). CFIR is a highly organized explanatory framework that identifies factors influencing implementation success from five domains: Characteristics of the outer setting, inner setting, innovation, individuals, and change process.

### Setting, Participants, Sampling, and Recruitment

This study was conducted in a large metropolitan tertiary hospital in Queensland, Australia. The Ambassador program was piloted in three surgical wards (two orthopedic wards and one general surgical ward). Patients or visitors over 18 years of age, nurses, staff from protective service and stakeholders who experienced the Ambassador program were eligible to participate. An email invitation and recruitment flyer were used for recruitment. Maximum variation sampling was used (Palinkas et al., 2015).

### Ambassador Program

The Ambassador program was piloted outside of business hours only (weekday evenings, nights, and weekends) with the support of QOVSU over 6 months in 2020. Ambassadors are Security Officers who operate using an innovative practice model compared with traditional security officer roles, by adopting a proactive client-centered approach and focusing on OV prevention. The description of the role is outlined in Table 1. The Ambassadors were recruited, supervised, and managed through Protective Services. They wore a nondescript hospital uniform—a polo shirt with Ambassador embroidered on it. The program was evaluated after the piloting period using surveys, interviews, and organizational data. This study focuses on reporting the qualitative findings from the program evaluation.

**Table 1:** Description of Ambassador Role

What	An Ambassador is a non-traditional Security Office embedded in a clinical setting. They wear a generic staff uniform that does not identify them as Security Officers.
Who	An Ambassador is a Security Officer with attributes aligned to the role, that is, advanced interpersonal communication skills, empathy, team player, calm under pressure, and non-confrontational.
How	Ambassadors are focused on the proactive engagement of patients and visitors to prevent violent and aggressive behaviors. They partner with staff to identify potential issues and employ behavior strategies to de-escalate or redirect persons of concern. An Ambassador does not replace the need for Security Officers. They do not engage in security responses unless there is an imminent threat and security is unavailable.
Where	Ambassadors are embedded in clinical areas identified as being at risk of occupational violence.
When and how much	Ambassadors are rostered at times when the risk of occupational violence is greatest. The number of Ambassadors per patient is based on an assessment of the risk and the geographical layout of the facility.
How well	Ambassadors are managed by the hospital Protective Services, which provides day-to-day supervision, training, and performance review.

### Data Collection

Written consent was collected from participants before any data collection took place. A semi-structured interview question guide (Table 2) was developed and piloted before use. The interview questions were developed based on the specific roles of the participants. An experienced qualitative researcher conducted the interviews in a private hospital room or over the telephone. After each interview, the interview schedule was adapted to explore emerging themes. Interviews were conducted until the researchers had a thorough understanding to generate insights (Thorne, 2016). Seventeen interviews were conducted and included in the data analysis.

**Table 2:** Interview Question Guide

Questions for staff, patients, visitors, and stakeholder participants
1. In what capacity have you experienced the Ambassador program (patient, staff, and stakeholder)?
2. Please describe a typical interaction with the Ambassadors.
3. How has the Ambassador program impacted you personally or others?
4. Do you think the initiative is appropriate/inappropriate for the ward/hospital? Why or why not?
5. What do you see as the primary role/essential functions/duties of the Ambassador? Is there anything about the Ambassador role you would change?
6. What qualities are needed in an Ambassador in your opinion?
7. Did you or anyone else express any concerns about the program? Would you support/against the program being continued? Why or why not?
Additional questions for staff participants
8. What might be some of the challenges sustaining the program in your ward? How might these be overcome?
9. What suggestions would you give to other wards or hospitals thinking of introducing the program?

### Data Analysis

Deductive analysis, informed by the Consolidated Framework for Implementation Research, was used. The interviews were transcribed verbatim and uploaded to Nvivo (Version 14). Transcripts were read in detail several times to identify similarities and differences between participants and patterns and themes. The review of the transcripts was interspersed with strategic periods of immersion in the literature as part of a process of synthesizing, theorizing, and conceptualizing (Thorne, 2016). The contents of the reflective journal were also utilized to enhance the understanding of the interviews. Following preliminary analysis, initial themes were discussed with participants, and their feedback was incorporated into further rounds of synthesizing, theorizing, and conceptualizing. Consensus on the themes was reached among researchers.

### Rigor

Several strategies were utilized to ensure the rigor of the study including using a reflective journal, the triangulation of data from multiple sources, and confirming initial themes and organizing framework with participants (Thorne, 2016). A reflective diary was used contemporaneously by researchers to document their thoughts, feelings, observations, and reflections throughout the research process (Olmos-Vega et al., 2022). These diaries serve a crucial role in ensuring the study’s rigor and transparency. Triangulation was achieved by gathering data on a theme from diverse sources with different perspectives. Subsequently, the themes and theme framework were discussed with participants in follow-up conversations, and their feedback was integrated into subsequent rounds of analysis.

## Findings

### Characteristics of Participants

A total of 17 people were interviewed. The length of interviews ranged from 45 to 90 minutes. There were ten nurses—four direct care nurses (N) and six from nursing management and leadership (NM). The remaining participants included one patient (P), one visitor (V), three protective service staff (PS), one internal (IS), and one external policy stakeholder (QNMU union representative; ES; Table 3).

**Table 3:** Participant Characteristics (n = 17)

Categories	Sub-categories		*N* (%)
Roles	Nursing	Clinicians	4 (24%)
		Managers/leaders	6 (35%)
	Protective services		3 (17%)
	Stakeholder	Internal	1 (6%)
		External	1 (6%)
	Patient/visitor	Patient	1 (6%)
		Visitor	1 (6%)
Gender	Female		9 (53%)
	Male		8 (47%)
Total			17 (100%)

Insights have been categorized into five themes: program characteristics, internal drivers, external drivers, individual experience, and implementation process.

#### Program Characteristics

The characteristics of the Ambassador program will be described from three areas including the role, location and function, supervision, training, and management. The Ambassador role includes the prevention of violence, aggression, and disruptive behavior:

An Ambassador is an embedded security officer who monitors the clinical environment for triggers, individuals whose behaviour deteriorates or shows a heightened sense of agitation. (NM 1)

The Ambassador role was seen as complementary to the traditional security role and not a replacement. If an Ambassador cannot de-escalate a situation, they withdraw and call security to ensure they can maintain a trusting therapeutic relationship with patients and visitors:

The ambassadors are hands-off. Ambassadors do not restrain patients because we want them to develop trust with the patients. (NM 2)

Wearing a polo shirt seemed to reduce the likelihood of a patient or visitor being triggered by the para-military style dress worn by Security Officers—security vest, utility belt, and radio:

Some patients on this ward have criminal histories. They’ve spent time dealing with police and the prison system, and sometimes the uniform can aggravate the problem. (NM 3)

The key features of the role described by participants were the proactive approach and collaboration with the health care team. Ambassadors were seen to partner with staff to identify potential issues and employ behavioral strategies to de-escalate or redirect persons of concern:

De-escalation is the most critical factor. They listen to the clinical handover to know who is agitated or going through withdrawal from alcohol or drugs. They pre-emptively act. (NM 3)

The Ambassadors were selected based on critical attributes essential for the role, including interpersonal communication skills, empathy, being a team player, staying calm under pressure, and being non-confrontational:

The crucial part of the Ambassador’s role is their ability to create connection, even with some of the most traditionally disconnected people. They can put themselves in other people’s shoes to understand why they might be getting aggressive, agitated, scared, or worried. (NM 1)

The preference was for the Ambassador role to be filled by a Security Officer upskilled with some clinical knowledge rather than a clinician upskilled in security. Having a Security Officer in the position was seen to remove the risk of the Ambassador being redirected to clinical duties in times of high demand:

I think having security as a background is essential. They are taught how to de-escalate. They are taught how to handle complex patients. (NM 2)

I want a skilled Security Officer who can build a connection with our patients, not an Assistant in Nursing that can be pulled away to provide patient care. (IS 1)

It was noted that not all Security Officers have the necessary qualities for the Ambassador role. Recruiting appropriate people for the position was seen as essential to the program’s success:

If you get the recruitment wrong, the model will not work. A recruitment process that ensures we pick people with compassion, empathy, and the ability to connect with our client group is critical. (NM 1)

The location and function of the role were important factors to consider. There was a belief among most participants that there were areas where the role would not be effective, but there was no consensus as to the restrictions:

I think there may be some environments where it will not work—for example, high acuity mental health. I don’t know if the Ambassadors can use the same approach with that client group. It may work in a low acuity mental health area. (ES 1)

It was acknowledged that not every ward needs an Ambassador. Nurses suggested that local OV data and risk profiles should be used to identify at-risk wards:

I don’t know that it’s necessary for every ward. I think that they need to look at your rates of OV. (NM 3)

Ambassadors were embedded in clinical units. Embedding the role close at hand made it easier for nurses to communicate their concerns and seek input:

Ambassadors are already there on the ward, proactive, talking to staff, patients, and family members. (NM 3)

The Ambassador monitors the environment to assist nurses in identifying individuals whose behavior is deteriorating or showing a heightened sense of agitation. Therefore, they could intervene to prevent escalation:

They [Ambassadors] recognise the triggers that nursing staff wouldn’t identify: Someone pacing or a flickering light aggravating someone—identifying triggers early and trying to address them before things escalate. (NM 3)

It was identified that supervision, training, and management of the Ambassador need to be considered. A close link between Protective Services and the Ambassadors was seen as critical for the program’s success:

The connection between our current security arrangements and the new role is key to success. (NM 5)

The Ambassadors noted that they tailored and adapted their role to the needs of the local practice environment in consultation with the ward manager and Protective Services management:

The Ambassadors have input into how the program runs—it’s a collaborative thing. I’m not the one telling them what to do. (PS 1)

In addition, the Protective Services Manager monitored the Ambassadors and their workload closely, particularly in the early initiation period:

They will not be there to prevent OV if they cannot effectively monitor the environment. (NM 4)

Participants suggested that a standardized training program for the Ambassadors should include topics such as trauma-informed care, a brief introduction to medical conditions that can manifest as behavioral challenges, therapeutic communication, mental health first aid, and Aboriginal or Torres Strait Islander health:

I would like to see a professional healthcare security role developed in TAFE or elsewhere, so there is a formal accreditation and qualification pathway. (NM 1)

#### Internal Drivers

The Ambassador Program was introduced due to the reported high level of OV in the hospital. Promoting the safety of nurses was one of the key drivers. The driver for this was staff safety and well-being. In our last survey, nursing staff were concerned about not feeling safe at work and not being supported. (NM 5)

The health care facility had prioritized OV prevention and had systems in place to implement and monitor mitigation strategies:

OV is on our organizational risk register. I report directly to the Executive Director on what is working and has made a difference. So, it’s high on the agenda. (NM 1)

A significant driver for the Ambassador program was the desire to find a more cost-effective approach to OV prevention other than security specials:

The leadership looked for another option to reduce OV other than constantly throwing money at it with security specials. In surgery, they realised that the overtime cost of security specialists meant they could afford their security in an Ambassador role. (PS 1)

The program had the commitment and active involvement of nursing leaders and managers at all levels of the organization:

We met with the DON (Director of Nursing) and two nursing directors . . . They were all very keen. They had a genuine interest in OV prevention. (IS 1)

#### External Drivers

Promoting a safe working environment was a statewide priority and well supported by multiple governments (QOVSU) and non-government bodies (QNMU):

It’s a high priority from a political point of view and the point of view of the Director-General [Health]. (IS 1)

The Union was actively involved in the pilot of the program. The nursing leadership acknowledged that they leveraged their support to help influence policymakers to support the program:

The Union was very supportive and a driver of the program. I kept them informed every step of the way. (NM 4)

The increasing rates of OV across the State had caused leaders to focus on OV. The violence toward health care staff was frequently covered in the local and State media which elevated the attention of stakeholders:

It makes it in the media when there’s violence against staff, particularly nurses. The public holds nurses in very high regard and always get headlines. (ES 1)

#### Individual Experience

OV was described to be one of the most concerning issues for direct care nursing staff because it had impacts on their professional and personal well-being:

Whether verbal or physical, constant exposure to OV has a cumulative effect. It builds up, and it can be something small that breaks them. They face it day after day for years, and then suddenly, there is the straw that breaks the camel’s back. (NM 6)

Participants reported having some initial reservations about the program because they had seen other OV initiatives implemented without success. However, after the trial, the program was widely supported:

There was some apprehension from the staff, who thought it was just another organizational band-aid—just another security officer in a different shirt. (NM 6)

The direct care nursing staff reported feeling respected and listened to because the organization invested in resources to reduce OV:

They [staff] talked about how safer they felt and how respected they felt that someone had listened to them, taken some action, and done something about it [OV]. (ES 1)

The nurses and managers saw the Ambassadors as a part of the health care team. They participated in the handover and helped to plan the management of patients at risk:

They will always speak to me [shift leader] about how the day has been and highlight any potential areas of concern. They also sit in on handover, so they know what is going on in the place. (NM 3)

The Ambassador’s strong relationships with patients and families were seen to benefit the team’s ability to provide person-centered care. The Ambassador had time to connect with patients, families, and visitors, which meant they could tailor their approach to specific individual needs. They also would share these insights with nursing staff to help them better plan patient care:

They get to know the patients and learn about their lives. They have the time to spend with them that, ideally, we would want nurses to have, but that will never happen. (IS 1)

Families and visitors reported that the Ambassadors helped them by providing support and advice or directing their questions to the appropriate team member:

They [Ambassadors] were just fabulous, absolutely brilliant. They would help me, talk to me. I had trouble with the taxi drivers to get back to my accommodation, and they even helped me sort that out. (V 1)

If I didn’t understand something, they would ask the nurses to come and explain what was happening. (P 1)

The nursing managers and leaders reported that they were prepared to allocate financial and human resources to programs such as the Ambassadors that ensure staff safety, well-being, and satisfaction with work. Knowing that the Ambassadors were present on the ward when they were not was a comfort to managers who reported that they worried about the safety of their nursing staff after hours:

It is good to know that after I go home and into the night duty, there is someone available that the team can call if they are concerned. (NM 3)

#### Implementation Process

The team lead described that before implementing the Ambassador program, she consulted widely with stakeholders, including staff, managers, executives, Protective Services, after-hours managers, and unions:

So we had everyone, we spoke about what we wanted the model to achieve and let them know it was happening. (NM 4)

Regular meetings and communication between team members were established to ensure effective communication and prompt decision-making:

We had fortnightly meetings. We met to ensure things were going to plan and discuss any concerns. [Protective Services Coordinator] would touch base regularly with the three Ambassadors and report back. (NM 4)

All project team members made it clear that interdepartmental collaboration between Protective Services and the clinical service was an essential ingredient to the success of the project. The project team developed strategies to ensure clear communication and teamwork during implementation and ongoing role monitoring:

This program worked because there was mutual respect between the clinicians and security. (PS 1)

Participants suggested that one of the lessons learnt from the implementation was the need to tailor communication based on the target group:

The biggest concern was how it was sold to the nursing staff. It was sold as a way to reduce Security and Assistant in Nursing specials. (NC 4)

At the end of the program, it was evaluated using multiple methods to comprehensively assess the effectiveness and usefulness of the program as a vital step of the implementation process:

We measured OV-related calls and duress alarm activations; OV-related workers’ compensation claims and workplace injuries; and financial costs and savings on specials. We collected data three months before and three months after the Ambassador pilot. (IS 1)

## Discussion

The results of the study are discussed below mapped to CFIR domains that are understood to affect implementation success (Powell et al., 2014).

### Program Characteristics

This project has provided a comprehensive overview of this pilot Ambassador program including a clear description of the role. It is vital to have a clear description of the role to mitigate the risk of program drift which may diminish its effectiveness. Program drift is when a program is unintentionally implemented without the core features that are essential for success (Cotterill et al., 2018). This project highlights the unique characteristics of the Ambassador role as an adjunct to the current security services . The practice model for Ambassadors differs from the traditional security role in several ways: Ambassadors wear a generic uniform, use proactive engagement methods and behavioral strategies to prevent and manage behaviors, and do not physically intervene unless there is an immediate threat to safety. Wearing casual clothes made them seen by staff and patients as extra support personnel who focus on behavioral management, which was considered vital to the successful implementation of the model as (WorkCover Queensland, 2022). It was agreed that individual attributes that best fit this role include advanced interpersonal communication skills, empathy, teamwork, being calm under pressure, and non-confrontational (WorkCover Queensland, 2022). Indeed, it was found that Ambassadors with good interpersonal skills could effectively advocate for patients with dedicated time and tasks.

### Internal Drivers

The CFIR recognizes that the inner and outer settings to which a program is introduced are active factors in its success and not simply a backdrop to implementation (Damschroder et al., 2009). Factors related to the inner setting include structural characteristics of the organization, culture, and readiness for change (Damschroder et al., 2009). This study identified several characteristics of the inner setting that supported implementation. First, there was a demand for change among the staff and management because the current level of OV was acknowledged as intolerable, and previous efforts to address it had limited success. Second, OV was an organizational priority (Queensland Health, 2016) with health care leaders’ who demonstrated a significant commitment to addressing it. Indeed, organizational readiness for change is vital as an inner driver for successful changes (Weiner, 2009).

### External Drivers

External factors that CFIR identifies as influencing implementation include policies, incentives, and the influence of other organizations and bodies (Damschroder et al., 2009). This study identifies several supporting factors. National and State work health and safety legislation such as the Occupational Health and Safety Law (Australian Law Reform Commission, 2011) was seen as a significant motivator for leadership to prioritize OV prevention. This legislation allows hospital leadership to be named and prosecuted for failing to prevent staff exposure to serious injury, including physical and psychological injuries caused by OV. Most influential was the fact that OV was a priority for the Queensland Health care service (Queensland Health, 2016), with a dedicated statewide OV Strategy Unit to lead the testing and scaling of prevention and management innovations. As suggested by Tan and colleagues (Tan et al., 2021), the increased reports of OV toward health workers and the frequent media coverage have heightened awareness among key health service and government stakeholders. In addition, Health service unions such as QNMU were influential in raising the profile of OV and advocating for increased resources and measurable improvements.

### Individuals Experience

Organizations are made up of individuals, and the success or otherwise of programs are ultimately established through the interplay between individuals and their effects on their teams, units, networks, and organizations on implementation (Keith et al., 2017). This study identified that OV prevention was seen as an important and useful intervention for nursing staff, managers, patients and visitors. The introduction of the Ambassador role made the nursing staff feel respected and listened to because it was a concrete action to address OV, which is likely to enhance nursing staff job satisfaction. Indeed, perceived organizational support to address OV promotes better job satisfaction, as well as reduces staff turnover and burnout rates (W. Liu et al., 2018). To nursing staff, the Ambassadors fill more than a security role; they are valued team members. They have time to connect with and observe patients, families and visitors and share insights with the care team to support patient and family-centered care. Greater social support is likely to mediate the adverse effects of OV in the workplace (Duan et al., 2019). Because of the collegial relationship and proximity, staff were more likely to seek an Ambassador’s assistance, meaning potential issues were dealt with earlier and incidents averted. For managers, having the Ambassadors present on the ward after they leave is a comfort for them as they worry about the safety of their staff after hours. In short, the Ambassador program was well accepted by nurses, managers, patients, and visitors.

### Implementation Process

The CFIR does not recommend or prescribe any implementation model or framework. However, the authors argue that, regardless of the approach used, success is most likely in the presence of four standard components of change: engaging, planning, executing, and evaluating (Damschroder et al., 2009). This study found extensive consultation with a broad selection of internal and external stakeholders before implementation was vital to the success of the program. The consultation aimed to build a shared vision for the program and gain the necessary buy-in and approvals. It was acknowledged for the project to be successful, there needed to be strong interdepartmental collaboration, which is also suggested in other research (Bond-Barnard et al., 2018). Strategies to ensure clear communication between Surgical Services and the Protective Service Unit were implemented to ensure effective project planning. The project leadership team was intentionally kept small to enable an agile change process that was adapted as needed based on data and feedback. Routinely collected incidence data and pre- and post-implementation staff surveys were used to monitor and evaluate the program.

### Strengths, Limitations, and Future Directions

A strength of this study is the use of a widely used implementation framework (CFIR) to promote consistent use of language, systematic analysis, and coherent organization of findings, which increases the validity of the study. A study limitation is the use of a single pilot site that had only trailed the program for 6 months at three surgical wards. Although these findings are essential for future implementation and spread of the program, they should not be interpreted as a guideline for implementing the Ambassador program. Future research should explore the value and acceptance of the Ambassador program by staff, patients, and visitors in other clinical environments with high risk of OV. More research is needed on the effectiveness, appropriateness, feasibility, and cost of the Ambassador compared to other security models.

## Conclusion

As a qualitative evaluation of the Ambassador program, this study provides valuable insight into the way nursing staff, patients, visitors, and stakeholders felt when engaging with Ambassadors in inpatient health care settings. The key lesson from the study is that the characteristics of the program, internal drivers, external drivers, individual experiences and the implementation process all impacted the success of implementation. Thus, the authors suggest future adoption of the program should involve an assessment of their local context and the utilization of a framework for designing the change plan and evaluating outcomes.

**Applying Research to Occupational Health Practice**

The introduction of the novel Ambassador program was seen as an innovative and valuable security model aimed at reducing the occurrence of occupational violence against nurses. The Ambassadors, characterized as friendly security personnel, proactively engaged in risk identification and intervention to prevent incidences of occupational violence. They were well integrated into the participating wards and received a positive reception from nursing staff, patients, and visitors. Innovative security models like the Ambassador program have the potential to contribute to the prevention of occupational violence against healthcare workers. Conducting future research to assess its effectiveness, feasibility, and cost-effectiveness would be beneficial in gaining a comprehensive understanding of this innovative role from various perspectives
